# Supplementary material for: From sequence to enzyme mechanism using multi-label machine learning
Source: BMC Bioinformatics. 2014 May 19;15:150. doi: 10.1186/1471-2105-15-150 (PMC4229970; doi:10.1186/1471-2105-15-150)
Supplement: Additional file 2 — Java code of ml2db. Additional file ml2db_code.tar.gz contains the Java source code to run the multi-label machine learning experiments and save the results to database. The code’s Javadoc is included. [file 1471-2105-15-150-S2.zip › additional file 2/ml2db/ml2db/doc/mulan/evaluation/measure/SubsetAccuracy.html]

SubsetAccuracy


JavaScript is disabled on your browser.


- Overview
- Package
- Class
- Use
- Tree
- Deprecated
- Index
- Help

- Prev Class
- Next Class

- Frames
- No Frames

- All Classes

- Summary:
- Nested |
- Field |
- Constr |
- Method

- Detail:
- Field |
- Constr |
- Method


mulan.evaluation.measure

## Class SubsetAccuracy

- java.lang.Object
- - mulan.evaluation.measure.MeasureBase
  - - mulan.evaluation.measure.BipartitionMeasureBase
    - - mulan.evaluation.measure.ExampleBasedBipartitionMeasureBase
      - - mulan.evaluation.measure.SubsetAccuracy

- All Implemented Interfaces:
  :   java.io.Serializable, Measure

  ---

    

  ```
  public class SubsetAccuracy
  extends ExampleBasedBipartitionMeasureBase
  ```

  Implementation of the subset accuracy measure. This measure is the opposite
  of the zero-one loss for multi-label classification.

  Version:
  :   2010.11.05

  Author:
  :   Grigorios Tsoumakas

  See Also:
  :   Serialized Form

- - ### Constructor Summary

    Constructors

    Constructor and Description

    `SubsetAccuracy()`
  - ### Method Summary

    Methods

    | Modifier and Type | Method and Description |
    | --- | --- |
    | `double` | `getIdealValue()` Gets an 'ideal' value of a measure. |
    | `java.lang.String` | `getName()` Gets the name of a measure. |

    - ### Methods inherited from class mulan.evaluation.measure.ExampleBasedBipartitionMeasureBase

      `getValue, reset`
    - ### Methods inherited from class mulan.evaluation.measure.MeasureBase

      `makeCopy, toString, update`
    - ### Methods inherited from class java.lang.Object

      `equals, getClass, hashCode, notify, notifyAll, wait, wait, wait`

- - ### Constructor Detail


    - #### SubsetAccuracy

      ```
      public SubsetAccuracy()
      ```
  - ### Method Detail


    - #### getName

      ```
      public java.lang.String getName()
      ```

      **Description copied from interface: `Measure`**

      Gets the name of a measure.

      Returns:
      :   the name of a measure.


    - #### getIdealValue

      ```
      public double getIdealValue()
      ```

      **Description copied from interface: `Measure`**

      Gets an 'ideal' value of a measure. The 'ideal' means, that the value
      represents the best achievable performance of a learner for an prediction of
      a multi-label task and associated true labels.

      Returns:
      :   the ideal value


- Overview
- Package
- Class
- Use
- Tree
- Deprecated
- Index
- Help

- Prev Class
- Next Class

- Frames
- No Frames

- All Classes

- Summary:
- Nested |
- Field |
- Constr |
- Method

- Detail:
- Field |
- Constr |
- Method
